# Bone augmentation for cancellous bone- development of a new animal model

**DOI:** 10.1186/1471-2474-14-200

**Published:** 2013-07-02

**Authors:** Karina Klein, Enrico Zamparo, Peter W Kronen, Katharina Kämpf, Mariano Makara, Thomas Steffen, Brigitte von Rechenberg

**Affiliations:** 1 Musculoskeletal Research Unit (MSRU), Equine Department, University of Zurich, Winterthurerstrasse 260, Zurich CH-8057, Switzerland; 2 Graduate School for Cellular and Biomedical Sciences, University of Bern, Bern, Switzerland; 3 Kuros Biosurgery AG, Technoparkstrasse 1, Zurich 8005, Switzerland; 4 Center for Applied Biotechnology and Molecular Medicine (CABMM), University of Zurich, Winterthurerstrasse 190, Zurich 8057, Switzerland; 5 Veterinary Anaesthesia Services - International (VAS), Zürcherstrasse 39, Winterthur 8400, Switzerland; 6 Division of Diagnostic Imaging, Vetsuisse Faculty, University of Zurich, Winterthurerstrasse 260, Zurich 8057, Switzerland; 7 Orthopaedic Research Laboratory, McGill University, 687 Pine Avenue West, Montreal, Qc H3A 1A1, Canada

## Abstract

**Background:**

Reproducible and suitable animal models are required for *in vivo* experiments to investigate new biodegradable and osteoinductive biomaterials for augmentation of bones at risk for osteoporotic fractures. Sheep have especially been used as a model for the human spine due to their size and similar bone metabolism. However, although sheep and human vertebral bodies have similar biomechanical characteristics, the shape of the vertebral bodies, the size of the transverse processes, and the different orientation of the facet joints of sheep are quite different from those of humans making the surgical approach complicated and unpredictable. Therefore, an adequate and safe animal model for bone augmentation was developed using a standardized femoral and tibia augmentation site in sheep.

**Methods:**

The cancellous bone of the distal femur and proximal tibia were chosen as injection sites with the surgical approach via the medial aspects of the femoral condyle and proximal tibia metaphysis (n = 4 injection sites). For reproducible drilling and injection in a given direction and length, a custom-made c-shaped aiming device was designed. Exact positioning of the aiming device and needle positioning within the intertrabecular space of the intact bone could be validated in a predictable and standardized fashion using fluoroscopy. After sacrifice, bone cylinders (∅ 32 mm) were harvested throughout the tibia and femur by means of a diamond-coated core drill, which was especially developed to harvest the injected bone area exactly. Thereafter, the extracted bone cylinders were processed as non-decalcified specimens for μCT analysis, histomorphometry, histology, and fluorescence evaluation.

**Results:**

The aiming device could be easily placed in 63 sheep and assured a reproducible, standardized injection area. In four sheep, cardiovascular complications occurred during surgery and pulmonary embolism was detected by computed tomography post surgery in all of these animals. The harvesting and evaluative methods assured a standardized analysis of all samples.

**Conclusions:**

This experimental animal model provides an excellent basis for testing new biomaterials for their suitability as bone augmentation materials. Concomitantly, similar cardiovascular changes occur during vertebroplasties as in humans, thus making it a suitable animal model for studies related to vertebroplasty.

## Background

Handling and injective properties of new biomaterials for vertebral bone augmentation can be tested in fresh cadaveric specimens, but *in vivo* experiments are required for proof of principle and safety reasons as the presence of blood flow could significantly alter the risk of leakage. Animals such as sheep, pigs, goats, calves, and dogs have been reported as models for several medical problems associated with the human spine [1], including bone defects [2] and kyphoplasty [3] in the lumbar area, although with a high complication rate. For vertebroplasty in sheep, mostly acute studies (euthanasia during anesthesia without recovery) were reported with the focus on cardiovascular complications such as pulmonary embolus formation. Cement leakage was followed within the systemic circulation, but not documented within the spinal canal as reported also in humans [4].

Pulmonary complications similar to those seen in humans have been described in vertebroplasty studies in sheep, where percutaneous [5], retroperitoneal [6,7] and open approaches [8-11] to the lumbar vertebrae were used. However, the minimally invasive surgical technique of percutaneous augmentation as commonly used for treatment of symptomatic vertebral compression fractures in humans, occurring secondary to osteoporosis or neoplasia [12-19], proved to be difficult to reproduce in the lumbar vertebrae of sheep. This is primarily due to the shape of the ovine vertebral body which is quite different and much more difficult to surgically access than in humans [3,20,21]. Even the standard surgical open approach to the lumbar area is complicated due to the large muscle mass in this area, the size of the transverse processes, the different orientation of the facet joints and mainly the slim, hour-glass shaped vertebral bodies. Even under fluoroscopic guidance, inadvertent penetration of the floor of the spinal canal with subsequent leakage of material into the dural space are serious complications and major limitations of this model [22]. In fact, this complication often leads to paraplegia and early termination of the experiment, although mostly these complications are never reported in the literature and only admitted in personal communications. A high percentage of the animals have to be euthanized immediately after recovery due to severe pain and clinical symptoms often require immediate termination of the experiments.

Apart from animal welfare reasons (suffering, numbers of animals used in the experiment), increased costs, high efforts, and unpredictable outcome necessitate a novel, simply reproducible and standardized animal model in sheep. In addition, the animal model preferably should reproduce the pulmonary complications ranging from no clinical signs to severe cardiac distress symptoms as seen in human and ovine vertebroplasty [10,16]. Moreover, a standardized and reproducible evaluation method to compare results of different studies should be available. We hypothesized that a femoral and tibial bone augmentation model in sheep would fulfill all these criteria.

## Methods

### Study design and experimental animals

For this novel experimental animal model the relatively dense cancellous bone of the distal femoral condyle and proximal tibia metaphysis was chosen as location for the application of radiopaque augmentation materials. A customized aiming device was developed that allowed repeatable, safe and standardized injections under fluoroscopic guidance.

For proof of this animal model 63 adult, female, Swiss alpine sheep with an average age of 2.7 years (2–5 years) and a body mass of 70.4 kg (49–99 kg) were used from consecutive studies focusing on different biomaterial formulations for augmentation (see Table 1). In all sheep, the same material was injected into the femoral condyle and proximal tibia of the same limb. Various radiopaque formulations of biomimetic materials based on a fibrin-scaffold were tested in comparison to sham controls. Since this manuscript is dedicated to the description of the animal model including evaluative procedures, the details of materials and results of bone enhancement will be published elsewhere (publication in preparation). Overall, 12 groups of materials were tested with 5–7 animals/group (Table 1). As controls sham surgeries were conducted in 5 sheep.

**Table 1:** Group distribution and CT analysis

**Group**	**Number of animals**	**Material**	**CT**	**Clinical signs of pulmonary embolism**
1	6	FS + MP1 + CA, with/without BM4	1	1
2	7	FS + CA, with/without BM4		
3	5	control/sham	2	
4	6	FS + MP2 + CA, with/without BM4		
5	6	FS + MP2 + CA, with/without BM4		
6	6	FS + CA, with/without BM4		
7	5	FS + MP2 + BM1	5	
8	5	FS + MP2 + BM2	4	
9	5	FS + MP2 + BM3	4	1
10	2	FS + MP2 + BM4		
11	5	FS + MP2 + BM5	2	1
12	5	FS + MP2 + BM6	1	1

Legend:

FS: Fibrin-scaffold.

MP1 or 2: Mineral powder 1 or 2.

CA: Contrast agent.

BM1-6: Biomimetic agent concentration 1-6.

with/without: One leg with and one leg without BM4.

This table shows the group distribution of all animals including the numbers of CT’s performed in each treatment group as well as the numbers of animals with cardiovascular complications occurring during surgery.

Due to the low morbidity and invasiveness of the surgical procedure, both hind limbs could be chosen, resulting in a total of four augmentation sites per sheep to inject material (n = 252 augmentation sites). For animal protection and welfare issues, a vertebral augmentation group as comparison to the new animal model was omitted due to the expected complications. (In a previous study in collaboration with an associated research group using an open and retroperitoneal approach to the lumbar spine, >80% of the animals had to be euthanized due to complications such as leakage into the spinal canal and fracture of the vertebral body).

All experiments were conducted according to the Swiss regulations of Animal Welfare and permission was granted by the local federal authorities (application # 7/2008).

The animals were acclimatized to the new environment approximately 2 weeks before surgery. A pre-anesthetic examination was performed including general clinical examination, hematology and chemscreen. Food was withdrawn 24 h before induction of anesthesia, while water was available ad libitum.

### Animal model

#### *Instruments/equipment*

**Figure 1:**
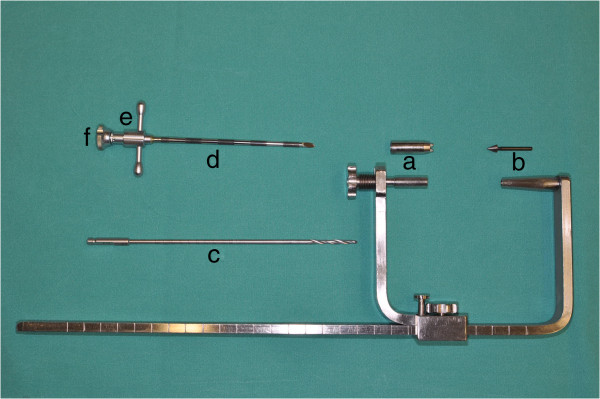
**C-shaped aiming device.** This photograph shows the c-shaped aiming device consisting of two adjustable arms with a mountable concentric outer tube with sharp teeth **(a)** and a mountable sharp tip **(b)** for device fixation. For application of biomaterial, a drill **(c)** and subsequently a 10G-vertebroplasty needle (needle **(d)**, handle **(e)**, and stylet **(f)**) were applied through the arm with the concentric tube **(a)**.

**Figure 2:**
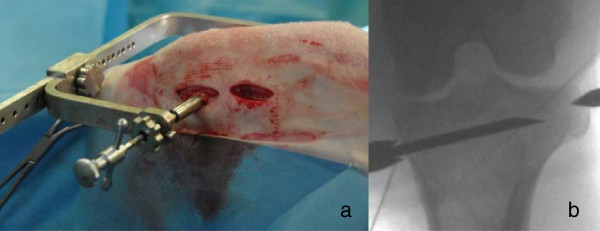
**Correct placement of the aiming device.** The aiming device was placed with the tube for the needle at the medial aspect and the sharp tip fixed on the lateral aspect of the tibia **(a)**. Fluoroscopy pictures were used to validate correct placement of aiming device and needle as well as subsequent material injections **(b)**.

In addition to the routinely used surgical instruments, a custom-made-aiming device was developed in order to increase reproducibility of surgical access and to standardize the injected area. The C-shaped aiming device allows exact positioning at the medial and lateral aspects of the tibia or femur including drilling in a given direction and at a predictable length (Figure 1). The aiming device consisted of two arms. The distance between the arms could be adjusted in order to fit the lateromedial axis of the stifle. A sharp tip is mounted axially on one arm while a concentric outer tube with sharp teeth is positioned on the other arm. The aiming device has to be placed with the tube for the drill hole at the medial aspect and the sharp tip fixed on the lateral aspect of the tibia or femur (Figure 2a). This positioning of the aiming device allows the insertion of the drill and ultimately the needle relatively parallel to the articular surface of the femoro-tibial joint. With the concentric tube and the distal sharp tip being in line, the suggested insertion trajectory for the needle can be validated using fluoroscopy in mediolateral and craniocaudal view (Figure 2b).

For the injection of augmentation material, a 10G (3.2 mm diameter) 100 mm-long special vertebroplasty needle (1394–1010, Optimed Medizinische Instrumente GmbH, Ettlingen, Germany) and an Optimed CementoRE gun (1392–0000, Optimed Medizinische Instrumente GmbH, Ettlingen, Germany) were used. The vertebroplasty needle consisted of three parts, needle, handle, and stylet (Figure 1).

#### *Anesthesia and analgesia*

Animals were premedicated intramuscularly with buprenorphine (Temgesic®, 0.01 mg/kg, Essex Chemie AG, Lucerne, Switzerland) and xylazine (Rompun® 2%, 0.1 mg/kg im, Streuli Pharma, Uznach, Switzerland). After approximately 30 minutes, a catheter (Vygonyle S® G14, Vygon GmbH, Aachen, Germany) was placed into one jugular vein and prophylactic antibiotics (Penicillin Natrium® 35′000 IU/kg i.v.; G. Streuli Pharma, Uznach Switzerland; gentamicin, Vetagent®, 4 mg/kg i.v., Veterinaria AG, Zurich, Switzerland) as well as a pre-emptive analgesic (Rimadyl®, 4 mg/kg i.v.; Pfizer SA, Zurich, Switzerland) were given intravenously. A booster for tetanus (tetanus serum Intervet, 3000 IU/sheep s.c.; Veterinaria AG, Zurich, Switzerland) was administered subcutaneously. Anesthesia was induced with diazepam (Valium®, 0.1 mg/kg i.v., Roche Pharma AG, Rheinach, Switzerland), ketamine (Narketan 10®, 2 mg/kg i.v., Vetoquinol AG, Belp-Bern, Switzerland) and propofol (Propofol®, 0.4-2 mg/kg iv, Fresenius Kabi, Stans, Switzerland) administered to effect. After laryngeal desensitization with lidocain spray (Xylocaine Spray 10%, 3 pumps of 0.1 mL, Astra Zeneca AG, Zug, Switzerland), the trachea was intubated and correct placement was confirmed by expired carbon dioxide monitoring (F_et_CO2). Anesthesia was maintained with a balanced anesthetic protocol employing administration of isoflurane (1-2%vol) in oxygen via an adult F-circuit (circle system, Intersurgical, Berkshire, UK) and a constant rate infusion of propofol (Propofol®, 0.5-1 mg/kg/h, Fresenius Kabi, Stans, Switzerland). All animals received intravenous fluids throughout the procedure (Ringer solution, 10 mg/kg/h). Cardiovascular monitoring parameters included electrocardiogram (ECG), heart rate, pulse rate and invasively measured blood pressures (systolic, mean and diastolic arterial) via an arterial catheter in an auricular artery.

#### *Surgical procedure*

The anesthetized sheep were placed in dorsal recumbency tilted at a 30° angle to the non operated side, with the hind limb slightly flexed and fixed at the surgery table. For the second leg repositioning was required. Aseptic preparation of the surgical field was performed routinely.

Two small incisions (1 cm) were performed at the medial aspect of the femoral condyle (directly above the femoral epicondyle) and at the metaphysis of the proximal tibia (at the distal end of the collateral ligament). Soft tissue was dissected down to the bone and the aiming device was placed with the tube for the drill hole at the medial aspect and the sharp tip fixed on the lateral aspect of the tibia or femur (Figure 2a). For fixation of the sharp tip on the lateral side only two stab incisions of the skin were needed, at the same level as the medial ones. Bleeding was controlled using an electrocautery.

Using fluoroscopy in two projection planes (mediolateral and craniocaudal), the right position of the aiming device and subsequent needle positioning could be validated and inaccurate injections avoided.

A drill hole with a diameter of 2.7 mm was created and followed by placing the special vertebroplasty needle with the tip ending in the trabecular portion of the femoral condyle (in parallel direction to the stifle joint surface) or proximal tibial bone (in slightly oblique direction to the fibula head).

**Figure 3:**
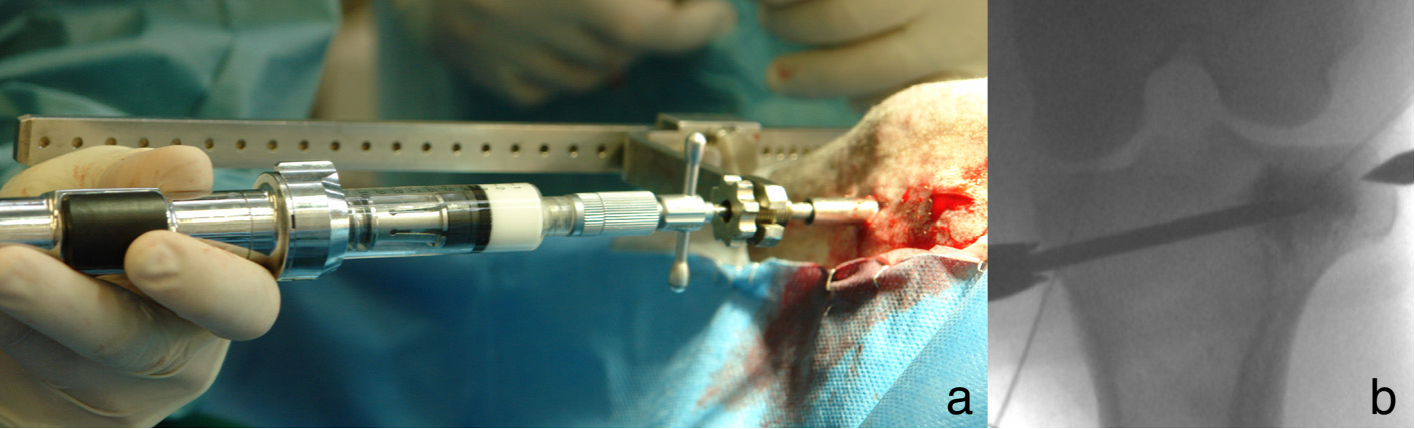
**Injection procedure.** Injections were performed with an Optimed CementoRE gun **(a)** and made in multiple steps under fluoroscopic control **(b)**.

The material injections were done in multiple steps under fluoroscopic guidance. Needle filling with biomimetic material was done slowly with the aid of the Optimed CementoRE gun (Figure 3). Subsequently, the gun was removed and a stylet was used to push the material through the needle into the bone, so as to provide exact quantities of injectate. If necessary a hammer was used to push the stylet and the material into the bone. The filling-clearing procedure was repeated three or four times, until a target of 2.7 mL was distributed within the intertrabecular space. Using fluoroscopy, leakage of the material into a vessel and thrombus formation was recorded and the amount of that material was estimated and documented.

Sham operations consisted of drilling and placing the needle as described above. The stylet was then inserted and removed three times without material injection.

At completion of the injection procedure, the vertebroplasty needle and the aiming device were removed and the insertion point was closed with a 3.5 mm diameter self-tapping screw (10 mm in length, Synthes, Oberdorf, Switzerland). The screw was used to determine the insertion point and the direction of the needle after sacrifice.

Closure of subcutaneous tissue was routinely done using resorbable suture material (Vicryl® 2–0, Johnson & Johnson Int., Brussles, Belgium) in a continuous fashion, while the skin was closed with a skin stapler (Appose ULV, United States Surgical). No further external stabilization was used.

After repositioning, the augmentation procedure of the contralateral limb was performed in identical manner. Immediately after surgery lateral and craniocaudal fluoroscopy images were taken to document the distribution of the injected material in two projection planes.

#### *Postoperative treatment*

After surgery and recovering from anesthesia, the animals were kept in small groups and their health status was checked twice daily. Food and water were offered ad libitum. Antibiosis and analgesia administrations were continued via the jugular catheter for 4 days using the same dosages as perioperatively applied. In addition, buprenorphine injections i.m. were continued every 4 hours for two applications to reduce postoperative pain.

After 21 days the staples were removed and the animals were released to pasture.

#### *Fluorescence labelling*

For a dynamic representation of new bone formation and remodeling, different fluorescence markers were subcutaneously injected at different time points. Calcein green (1 ml/kg s.c.; Fluka AG, Buchs, Switzerland) was applied after 4 weeks, xylenolorange (1 ml/kg s.c.; Fluka AG) three days before sacrifice.

### Evaluation

#### *Computer Tomography Angiography*

Computer Tomography Angiography (CTA) of the thorax was performed in 19 sheep either after cardiovascular complications developed during surgery indicating pulmonary embolism or in selected cases to visualize the potential occurrence of pulmonary embolism caused by bone marrow/fat particles or material deposition. The CT study was performed with a multi-row unit using 40 rows, slice collimation of 0.6 cm and a pitch of 1. The contrast medium dose was 700 mg/kg of an iodinated high-osmolar contrast medium (Telebrix®, 350 mgI/ml, Guebert, Zurich, Switzerland). The volume of embolized material was calculated using an automated region of interest propagation technique with an upper attenuation threshold of 5000 Hounsfield units (HU) and lower threshold of 300 HU.

#### *Specimen preparation for evaluative procedures*

After sacrifice, the tibia and femur were extracted for final analysis. Augmentation sites were examined macroscopically and digital pictures were recorded (Nikon Digital Camera D5000, © 2009 Nikon Corporation). Subsequently the bones were radiographed using a Faxitron (Faxitron X-Ray Systems; Hewlett Packard, Mc Minnville Division, OR) to visualize the detailed bone structure and detect remaining augmentation material.

To provide a standardized evaluation process, an appropriate harvesting method was designed to locate the area of interest for histological evaluation.

**Figure 4:**
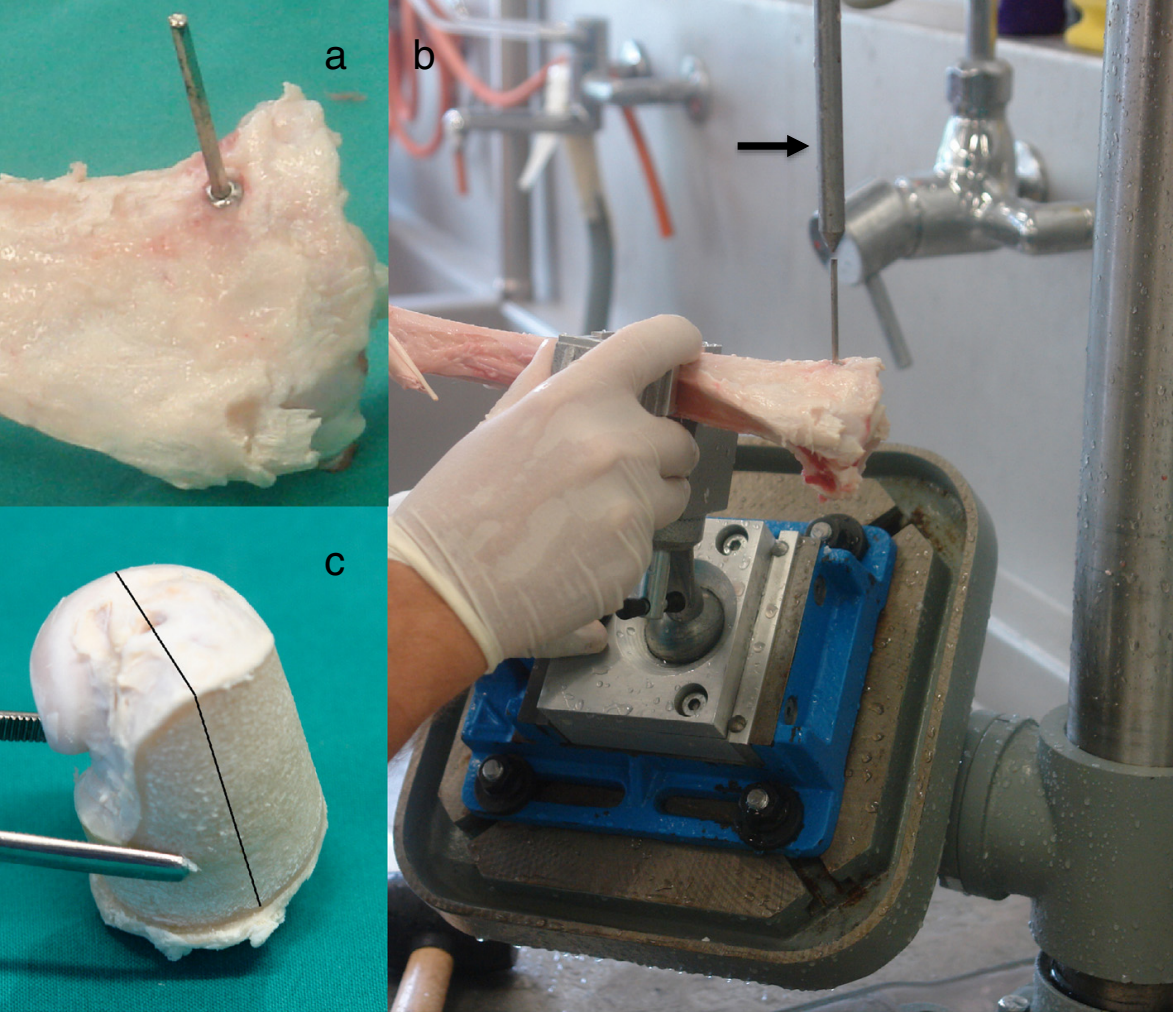
**Harvesting method.** To provide a standardized evaluation process and locate the area of interest, an appropriate harvesting method was designed: Using a custom-made screw the original direction of the needle could be established **(a)**. A custom-made holder allowed fixation of the bone to a drill press table aligning the original needle path with the axis of a diamond-coated water-cooled core drill **(b)**. The black arrow marks the pin pointer used for defining the drill axis. After screw and pin pointer removal, the core drill was mounted on the drill press and a 32 mm diameter bone cylinder was cut **(c)**. The black line in Figure **c** marks the cutting mark for histology sections with original needle insertion point and needle path in the center.

By fitting a custom-made axially lined up metal pin pointer into the head of the screw, the original direction of the injection needle could be established (Figure 4a). The bone was fixed to the drill press table with a custom-made universal joint holder. By manipulating the holder, the appropriate position could be found by ultimately aligning the original direction of the injection needle with the central axis (Figure 4b) of the drill press. After screw and pin pointer removal, the 32 mm diameter, water-cooled diamond core drill was mounted on the drill press and an accurate bone cylinder was cored with the original needle path matching the axis of the cylinder (Figure 4c). A mark was made with a band saw on the cortical bone from the entry point of the needle in distal direction. This allowed consistent positioning of the sample for μCT scanning and served as a reference for the cutting of the histology slices.

All bone samples were fixed in 40% ethanol for 1 week, followed by a series of ethanol dehydration (50-100%) within 6 days. Once the specimens were fixed in 70% ethanol, μ-CT analysis was performed by b-cube AG (Bio-Technopark, Schlieren-Zurich, Switzerland).

After μ-CT analysis and complete dehydration all samples were degreased in xylene and subsequently infiltrated in liquid polymethylmethacrylate (PMMA). Polymerization was carried out in plastic molds closed with a lid and kept at 4°C for 1 week, thereafter in a water bath at room temperature until polymerization occurred. Finally the plastic molds were placed in an incubator (37°C) uncovered to complete hardening of the samples.

*Ground sections* (300 μm) and *native fluorescence sections* (350 μm) were cut using a special band saw (EXAKT® Band System 300/301, EXAKT® Apparatebau GmbH &Co KG, Norderstedt, Germany). The blocks were cut lengthwise the needle path. The needle path, the tip of the needle, and surrounding tissue defined the region of interest. Before mounting (Cementit® CA 12, Merz + Benteli AG, Niederwangen, Switzerland) the sections on plastic slides (ACROPAL, Maagtechnic, Duebendorf. Switzerland), microradiographs were taken in the Faxitron (27 kV, 11 s; Fuji Photo Film Co Ltd, Tokyo, Japan). Ground sections were polished (Exakt® Mikro- Schleifsystem 400CS, Exakt Apparatebau GmbH, Norderstedt, Germany) and surface stained with toluidine blue. The native sections for fluorescence were mounted on pellucid, acrylic plexiglas slides (Maagtechnic, Duebendorf, Switzerland) and wrapped in aluminium foil to protect slides from bleaching.

For the *thin sections* the area of interest of the remaining bone blocks, comprising the tip of the needle and surrounding tissue, was cut into a smaller segment and polymerized in special customized Teflon forms (D. Nadler, JOSSI AG, Islikon, Switzerland) at room temperature. For hardening, the teflon forms were put in the incubator at 37°C. Thereafter, blocks were mounted on plastic frames and cut using a microtome (Leica® RM 2155, Leica Instruments GmbH, Nussloch, Germany). After placing the sections on glass slides, they were deplastified with Methoxyethyl-acetate (Merck AG, Switzerland) and stained with either toluidine blue or von Kossa/McNeil.

#### *Histological evaluation*

The standardized cutting procedure of all samples along the original needle path ensured comparability of the results obtained for the different treatment groups. The area around the tip of the original needle, where most of the augmentation material was injected, was identified as region of interest for analysis.

*Ground sections* were evaluated histomorphometrically (Leica Qwin®, Leica Quips®, Leica, Glattbrugg, Switzerland) to quantify the percentages of new bone, old bone, granulation tissue, and residual augmentation material within the area of interest.

**Figure 5:**
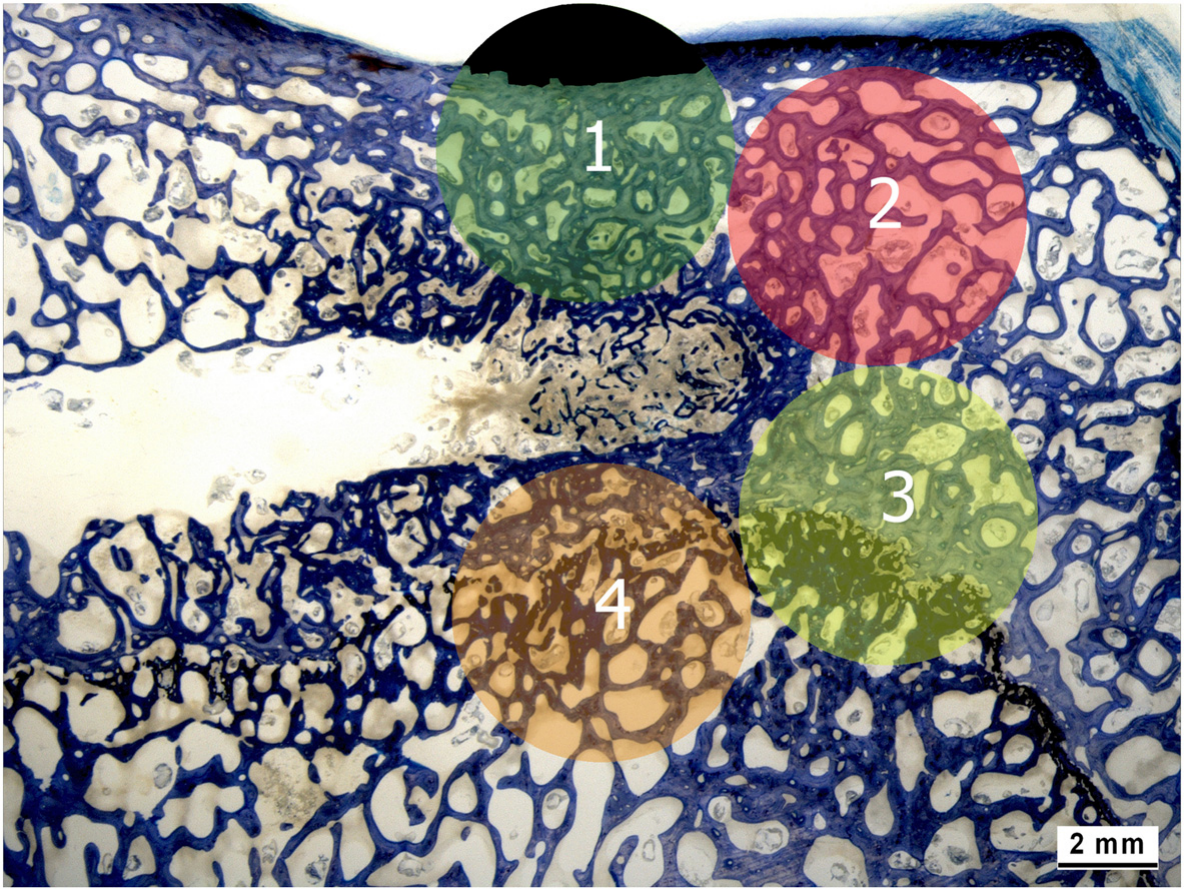
**Histomorphometrical evaluation.** Histomorphometry was used to quantify the percentage of new bone, old bone, granulation tissue, and residual augmentation material within the area of interest. For standardization of the evaluated area four circles of 6 mm in diameter were chosen around the tip of the needle. Cortical bone area was excluded from the analysis (black area in circle 1). The rest of the circle was considered as 100%. Circles including growth plate were completely excluded from the analysis (circle 3 + 4).

First, the ground sections were captured with the macroscope (Leica Z6 APOA, Leica DFC 420C, Glattbrugg, Switzerland) as digital images in TIF-format using a 5.6-fold magnification. To standardize the evaluated area four circles of 6 mm in diameter were chosen around the tip of the needle (Figure 5). If the circles contained cortical bone it was excluded from the region of interest, since more old bone with high density is present in the cortex. The rest of the circle was considered as 100%. Circles including the growth plate were excluded from the analysis, since a higher rate of new bone was present independently of the applied material.

*Thin sections* were semi-quantitative evaluated using a microscope (Leica® DMR, Glattbrugg, Switzerland) in a ten-fold magnification. Scores were given for presence of macrophages, mononuclear cells, osteoclasts, multinucleated foreign body cells, debris of bone, isles of not calcified osteoid, agglomeration of mononuclear cells, percentage of osteoid seam, osteoid thickness, and material in bulk. Three power fields per section were assessed with two peripheral and one central in the injected area. For macrophages, mononuclear cells, and fibroblasts scores were given in percentages of cells per power field (0 = 0%; 1 = 1-25%; 2 = 26-50%; 3 >50%). For multinucleated foreign body cells and osteoclasts absolute numbers were counted per power field (0 = 0; 1 = 1-5; 2 = 6-10; 3= >10 cells).

*Fluorescent sections* were evaluated at 1.25× magnification with a special fluorescence microscope equipped with different filters appropriate for the used dyes (Leica® DM 6000B, Leica Microsystems CMS GmbH). To achieve one adequate picture including the whole area of interest centered at the tip of the needle, 30 images had to be taken and merged together to one using a compatible camera (Leica®DFC 350 FX, Leica Microsystems CMS GmbH, Mannheim, Germany) and a specific merging software (Leica® Microsystems CMS GmbH, Mannheim, Germany).

Focus was placed on differences of dye integration between groups as well as at different time points (calcein green at 6 and xylenol orange at 12 weeks).

A semiquantitative evaluation of the recorded images was performed scoring the percentage of each fluorescent dye in the area of interest (considered as 100%) (0 = 0%; 1 = 1-25%, 2 = 26-50%, 3= >50%).

#### *Micro CT evaluation*

All samples were measured with a micro-computed tomography system (μCT 40, Scanco Medical AG, Brüttisellen, Switzerland). Semiautomatic masks of sphere shells in different sizes were performed centered at the original position of the tip of the needle to evaluate bone volume density and trabecular thickness of these spheres as well as the residual radiopaque material. To measure only the trabecular bone area, the needle path and the cortical bone were excluded from the analysis.

## Results

### Animal model

A total of 63 sheep were treated with the tibia and femur augmentation model. Sixty-two sheep completed the 12 weeks follow-up period successfully. One animal had to be euthanized 6 weeks after surgery due to the presence of a malignant lymphoma in the wall of the urinary bladder, unrelated to the study treatment.

Augmentation could be achieved successfully in all 63 animals and after a learning curve initially 245/252 augmentation sites were highly reproducible and without complication. In 7/252 needle positioning problems occurred (see below). The overall surgical procedure for each hind limb was between 30–40 minutes. In addition, the minimally invasive surgical procedure led to immediate recovery and minimal physical stress. After a short recovery period due to anesthesia (e.g. 30 minutes), the sheep rose and moved around without lameness. During the 12 weeks follow up, no signs of inflammation, wound infections, lameness or other discomfort were noticed in any of the 62 remaining sheep. Furthermore, the sheep exhibited no pain response following palpation of the injected sites.

The surgical technique using the aiming device and fluoroscopic imaging were particularly useful to inject the material into a standardized anatomical location. Under fluoroscopic guidance during surgery, it could also be verified that consistent amounts of biomaterials were injected into the trabecular bone and distributed in a sphere-like shape around the tip of the needle. This distribution and needle placement could also be verified using the evaluative procedures. μCT images, microradiographs, indicated very consistent and standardized injection areas (see below).

Due to the high density of the trabecular bone at the injection site, the needle could not be introduced with manual pressure as it is described in human medicine. Instead, a hole was drilled and the needle pushed into the trabecular bone by using a mallet. As mentioned above, needle-positioning problems occurred in only 7/252 injection sites. In two of these cases, the needle direction was slightly different than the original drilling path resulting in a second hole at the tip of the needle, although without associated complications. In the remaining five of these seven cases, the needle positioning was assumed to be in the correct place based on fluoroscopy. However, upon injection a small amount of the biomaterial was identified within the joint space in two cases and within the periarticular soft tissues in three cases. When leakage of material was discovered, the reposition of the needle was attempted in order to inject in the predefined area. In three cases there was no additional leakage during the following injection. In two cases the injection was cancelled due to persistent leakage during the additional attempts.

To inject the augmentation material along the small diameter of the vertebroplasty needle into the dense intertrabecular bony space, high injection pressure was necessary. During the injection procedure the viscosity of the augmentation materials increased depending on the duration of the procedure. For the second and third injection of each augmentation site, a mallet was required to cautiously push the stylet and force the material along the needle into the bone.

The radiopaque materials were visualized without problems during injection in 208 augmentation sites. In 24 injection sites of 6 sheep of the same group, the material could not be identified with fluoroscopy due to inadequate radio-opacity of the augmentation material. This augmentation material was considered not suitable for injections under fluoroscopic control. In the 20 sham control sites (5 sheep) no material was actually injected and thus, could not be visualized. Leakage of augmentation material was observed in 90 out of 208 injections (43.3%). No differences between the various materials could be allocated. Out of those 90 cases, leakage into the venous circulation occurred in 54 injections (26%), whereas leakage into the medullary cavity was observed in 31 injections (14.9%). Leakage into the periarticular soft tissues was detected in 5 additional cases (2.4%). A higher number of leaks during the injection procedure occurred in the tibia (60%) than in the femur (40%), especially for the second and third injections at the injection sites where higher pressure had to be applied for injection. Despite the high number of biomaterial leaks observed with fluoroscopy, no clinical symptoms indicative of cardiovascular and/or pulmonary distress were recorded in 59 sheep (93,7%). In four animals, cardiovascular complications occurred during surgeries including sudden decrease in arterial blood pressure and end-expired CO_2_ by approximately 50%. In those four animals, CT angiography of the thorax revealed embolization with augmentation material and bone marrow, and fat particles. All four animals recovered without additional complications and completed the 12-week follow up successfully. Based on this experience, a randomized and prophylactic CT analysis of subsequent 15 sheep was performed, which demonstrated lung embolization in 13/15 animals undergoing augmentation with injection of biomaterial. In the other two animals (2/15), which belonged to the control group, no embolization was found. Out of the 17 sheep treated with augmentation material and demonstrating embolization, 7 sheep (41.2%) showed embolism caused by bone marrow and fat particles as well as material deposition, while 10/17 sheep (58.8%) revealed embolism due to material only. Overall 202 emboli were found in 17 sheep treated with augmentation material. They occurred in the majority of the cases in the subsegmental artery within the lung vasculature, for bone marrow respectively fat particles (12 emboli) and material (156 emboli), but there were emboli in the segmental (6 fat emboli/24 material emboli) as well as in lobar artery (1 fat emboli/3 material emboli), although in lower incidence with the latter.

Another surgical complication was the breaking of the needle during the extraction procedure from the bone. The needle broke, or rather dismantled at the glued junction between needle and handle. In 4 of 14 cases, the broken part of the needle could not be removed and remained in the bone so that the insertion of the self-tapping screw was not possible. However, no further complications resulted from this event, although the absence of the screw made the coring procedure after sacrifice less precise, and all bone cylinders contained the entire area of interest.

### Evaluation

The harvesting process of the tibia and femur at sacrifice were uneventful. μCT, microradiography, and histology were excellent tools to verify needle placement and material distribution within the local anatomic sites. On the one hand, material deposition was still visible and on the other hand bone remodeling alongside the original drill hole and the tip of the needle clearly identified the location.

Due to early termination of one sheep, only 248 samples were further processed for evaluation. The harvesting process itself, in terms of drilling the 32 mm bone cylinder and preparing the area of interest for evaluation, proved to be reproducible and guaranteed a highly standardized analysis in 243/248 samples. Three out of 248 samples had to be excluded from the histological evaluation since the tip of the needle was not centered in the cylinder so that the ground sections were cut in the wrong layer. In the other two, no injection was performed due to needle positioning problems. All other samples could be embedded, cut and prepared for analysis without further complications.

**Figure 6:**
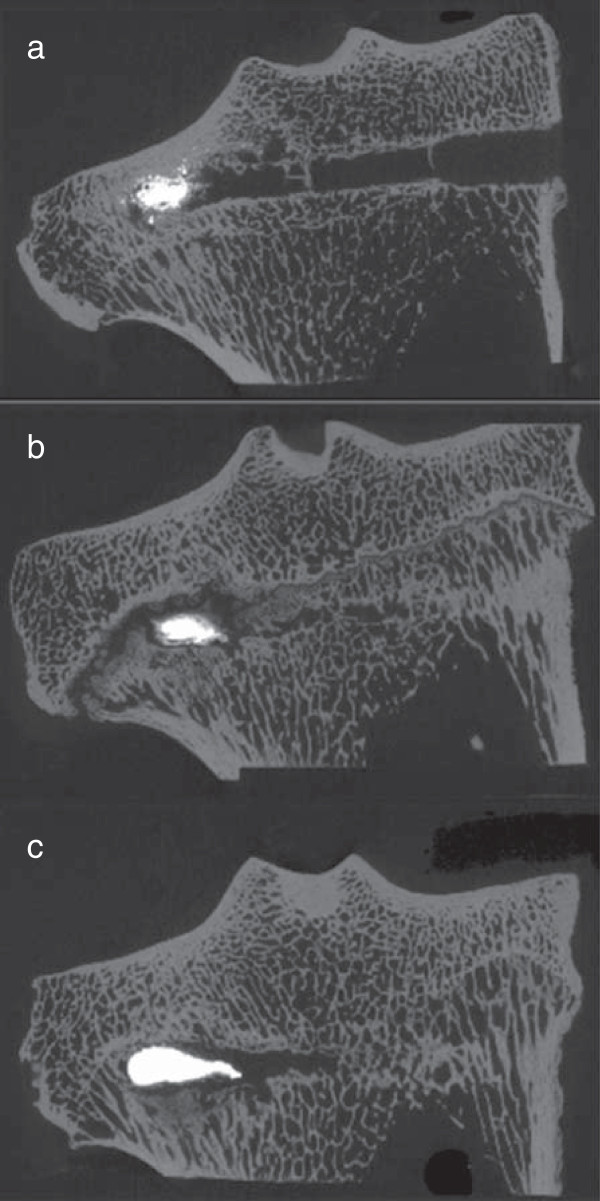
**Micro-CT images.** Micro-CT images confirmed the suitability of the surgical method with respect to the consistent placement of the needle in the desired trabecular bony space. Due to the presence of an open physis in the proximal tibia in some animals, the injection resulted in deposition of biomimetic material in the trabecular bone within the epiphysis **(a)**, the physis **(b)**, or in the metaphysis **(c)**.

Needle placement could be visualized in the μCT images due to the fact that the original needle path was not yet healed. μCT confirmed the suitability of the surgical method with respect to the consistent placement of the needle in the desired trabecular bony space. Due to the presence of an open physis in the proximal tibia in some animals, the injection resulted in deposition of biomimetic material in the trabecular bone within the physis, in the epiphysis or in the metaphysis (Figure 6). In contrast, injections in the femur resulted in consistent deposition of augmentation material within the distal femoral epiphysis. The μCT evaluation of the control animals showed a relatively high bone volume density (Bone volume/total volume) with thick trabeculae for both, the femora and the tibiae with a 10% higher bone volume density for the tibia metaphysis (approx. 35%) than for the femora (approx. 25%).

**Figure 7:**
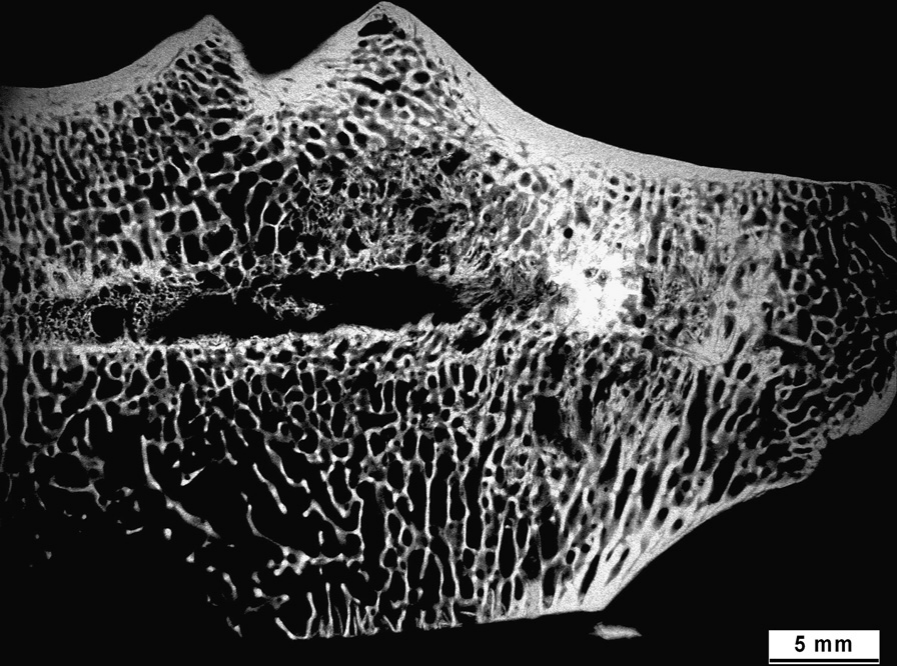
**Microradiograph image.** This figure shows a microradiograph image of a ground section: Stage of bone calcification in the area of interest and adjacent to remaining biomaterial could be shown.

**Figure 8:**
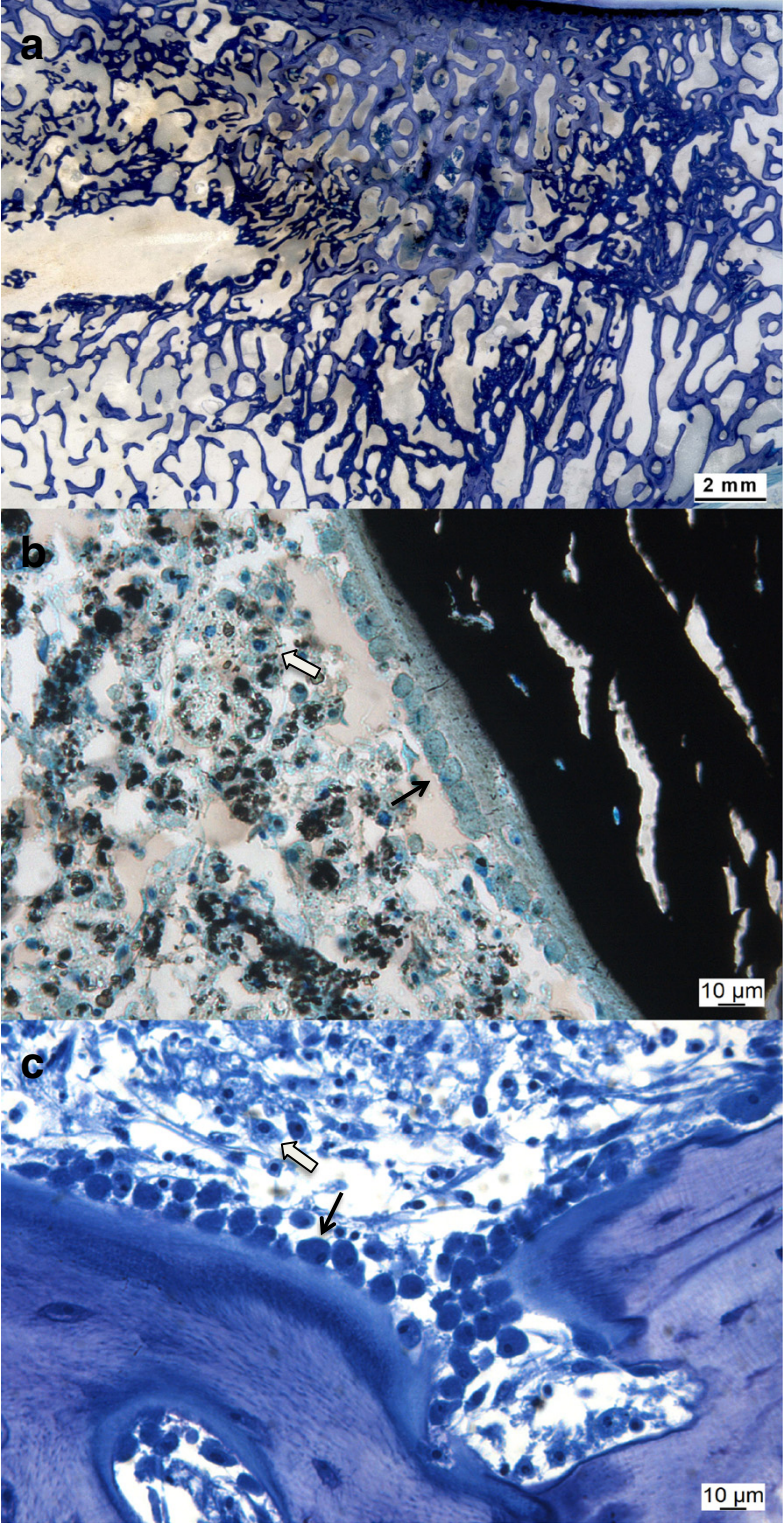
**Histology samples.** Ground sections (approx. 300 μm, surface stained with toluidine blue) were well suited for assessment of new bone formation, material resorption and histomorphometrical evaluation (Figure **a**), while thin sections allowed assessment of cellular reactions e.g. osteoblast activation (Figure **b**, **c**, thin black arrows) and biomaterial degradation through macrophages (Figure **b, c**, thick white arrows).

With the microradiograph images of the ground sections, the stage of calcification of the bone samples within the area of interest and adjacent to remaining biomaterial could be identified as too could the original and standardized needle positioning (Figure 7). While the ground sections were well suited for the assessment of new bone formation, material resorption, and histomorphometrical measurements (Figure 8a), thin sections enabled distinctions to be made between cellular reactions such as osteoblast activation, inflammatory responses or degradation and elimination of biomaterials through macrophages (Figure 8b, c).

**Figure 9:**
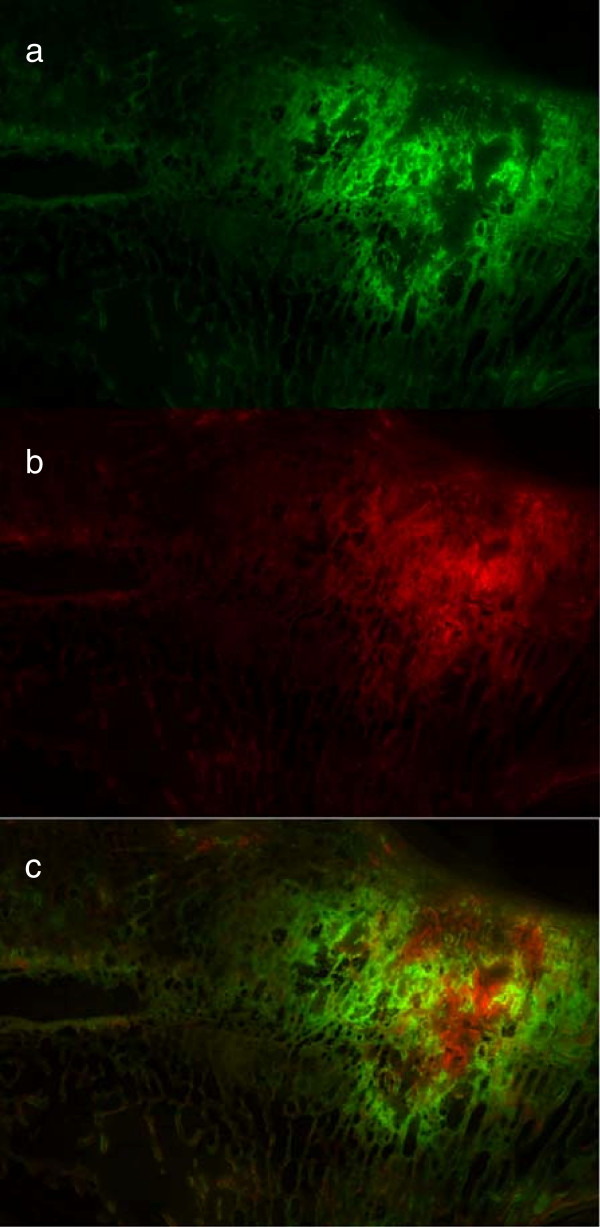
**Fluorescence section.** These fluorescence images show the bone activity grade at two different time points according to the integrated fluorescent respectively. Figure **a** shows the activity grade at 6 weeks post op (Calcein green), Figure **b** at 12 weeks post op (Xylenol orange), and in Figure **c** both time points are merged together to one image.

The fluorescence sections represented the area of bone activity at six weeks post surgery (Figure 9a, green colours) and at twelve weeks post surgery (Figure 9b, red colors) and demonstrated the sphere-like shape distribution of the biomaterial. A less colored area was observed in the middle of the injected region, corresponding to the biomaterial degradation process followed by a red and a green area towards the periphery. This pattern indicated a radial kinetic of new bone formation while biomaterial was being degraded from the periphery towards the center of the injected region (Figure 9).

## Discussion

In our study, a femoral and tibial bone augmentation model was successfully established and validated using 63 sheep as an alternative to vertebroplasty in the lumbar area of sheep. In comparison to the lumbar vertebroplasty procedures in sheep, the femoral-tibial augmentation model proved to be safe, easy and quick to apply, and additionally showed a high level of standardization including the harvesting of core samples for histology and the histology evaluation. The surgical procedure was limited to 30–40 minutes per side, which was significantly shorter than pilot studies (unpublished data) with lumbar vertebral augmentation in sheep, which lasted for several hours. More importantly, the procedure eliminated severe complications (massive spinal cord injuries) and reduced animal pain and suffering. Moreover, the animal model proved to be adequate to study pulmonary embolism and systemic changes as sequelae to vertebroplasty as they occur in humans.

The animal model presented served well to evaluate new biomimetic materials for their osteoinductive, osteoconductive, and degradation properties as bone augmentation materials. The advantage was that the test-materials could be injected with high accuracy and repeated success within the inter-trabecular spacing of the intact femoral condyle and proximal tibia. This surgical technique required a specially developed aiming device and fluoroscopic imaging. Both are also required for (percutaneous) vertebroplasty in humans. The application and visualization of the injected material in the region of the stifle joint in sheep is much easier compared to the lumbar area, where visualization is impaired by overlying structures (intestines, rumen, transverse processes, etc.). The absence of the third plane using two-dimensional fluoroscopy has to be taken into consideration during needle positioning and injection procedure to avoid injections out of the predefined area, which occurred only in 5/252 needle placements. Solid knowledge of how the trabecular area is distributed within the proximal tibia and experience of the surgeon helps to avoid injections into the bone marrow and/or penetration of the caudo-lateral cortex of the tibia with the needle.

When comparing the results of Hildebrand *et al.*[23] retrieved from human bone samples with those of Rubin *et al.*[24] from sheep bone, it becomes evident that trabecular bone density is much higher in sheep than in human bone. This comparison of previous findings of other research teams is in alignment with the results of our study presented here. Hildebrand *et al.* measured for samples of lumbar human spine (2. and 4. vertebra) a low bone volume density (8-9%) with small trabeculae, whereas in the current augmentation sheep study bone volume densities between 25-35% for tibiae and femura and even higher values in the study of Rubin *et al.* for medial femoral condyles (49.1 ± 7.1%). This high bone density and thick bone trabecula make the needle placement and the injection procedure more challenging in sheep (personal surgical experience). To place the needle for percutaneous VP in the osteoporotic, brittle human bone, it only has to be tapped with a mallet to engage the cortical bone and afterwards can be pushed forward with manual pressure into the right position [25]. In contrast, in sheep, a hole had to be drilled first to be able to push the needle forward into the trabecular bone with the aid of a mallet. This procedure weakened the needle and caused breakage at the glued junction between needle and it’s handle during the extraction procedure of the needle in 14 cases. Due to the fact that the needle could not be extracted in only four cases, and no further complication resulted from this event, the needle type was not changed during this study. For future studies, the use of another type of needle, e.g. with a melted junction (not commercially available at the moment) could be taken into consideration to avoid needle breakage.

During the injection procedure, the viscosity of the augmentation materials increased depending on the duration of the procedure. As such, forced injections under high pressure using a mallet were often necessary for the second and third injections of each injection site along the small diameter of the vertebroplasty needle into the dense intertrabecular bony space of the tibial and femoral bone. This could have led to a higher risk for material extravasation as well as bone marrow and fat particles being forced out into the systemic circulation.

The higher percentage of material leakage detected in the tibia as compared to femur, could be explained by the approximately 10% higher trabecular bone density and thicker trabeculae in the proximal tibia metaphysis than in the femoral condyle, which may have produced higher resistance to the augmentation material. Pulsed jet-lavage of bone marrow and fat was reported to improve injection and lessen the risk of emboli [26]. However, in our hands it did not facilitate injection in a preliminary study and therefore, the fragmented injection technique was invented and applied throughout the study. Although it may not completely mimic vertebroplasty in osteoporotic human bone, it may be considered close enough to test substances in sheep for later use in human bone. During vertebroblasty in humans, leakage of bone cement is also reported as the most common complication [25,27]. Therefore, this animal model could provide important information about the material properties if leakage occurs during the injection procedure. Due to the absence of chest radiographs or CTs after vertebroplasty on asymptomatic human patients, cement leakage and pulmonary emboli occurrence secondary to vertebroplasty may often be underestimated [16]. In the presented study in sheep, CT angiography of 19/63 sheep revealed a high number of asymptomatic pulmonary emboli (13/19 sheep = 68.4%) confirming the opinion of Hulme *et al.* that CT is the best method to detect cement leakage and should be used to report all leaks, including those without clinical signs [16,28,29].

Another consequence of the forced injections of augmentation material was microdamage and microfracture of trabecular bone, which occurred centrally to the injected area, and could be seen in the histology sections as well as in the fluorescence sections. This trabecular damage may be less in human osteoporotic bone due to reduced bone quality and quantitiy (wider trabecular spaces). Alternatively the diminished bone quality may also facilitate microfractures within the trabecular structure, thus resulting in a similar situation as encountered in sheep. Since osteoporotic bone is weaker, but not impaired in proliferation [30], remodeling activities may be similar to those present within our sheep model. As a consequence, a resorption zone around the injected area developed in some cases unrelated to the injected material itself.

## Conclusions

This tibia and femur augmentation sheep model proved to be a suitable animal model for screening studies of new biomaterials intended for bone augmentation of trabecular bone and enabled analysis of their biodegradable, biocompatible and osteoconductive properties. It also proved to be adequate for the study of the sequelae of material leakage into the circulation, e.g. pulmonary embolism and following cardiovascular changes.

Overall, we consider it a safe model for the experienced surgeon, resulting in reduced animal suffering and could serve as an excellent basis for testing bone inducing or enhancing materials for vertebroplasty.

## Competing interests

The authors declare that they have no competing interests.

## Authors’ contributions

KKL was the PhD Student and involved in all aspects of the study including writing the manuscript. EZ was involved in developing the model and material. PWK was the anesthetist and was involved in demonstrating embolization. MS was the anesthetist and was involved in demonstrating embolization. KK performed histology and was involved in developing bone sampling. MM was the radiologist and performed all CT including evaluation. TS designed the device and was involved in all aspects of the study. BvR was the head of the experiment, supervisor of PhD Program and was involved in all aspects of the study. All authors read and approved the final manuscript.

## Pre-publication history

The pre-publication history for this paper can be accessed here:

http://www.biomedcentral.com/1471-2474/14/200/prepub
